# Structure and Function of the Achilles Tendon and Plantarflexors 1 Year Following Achilles Tendon Rupture in the United Kingdom: A Cross‐Sectional Study

**DOI:** 10.1002/jfa2.70134

**Published:** 2026-02-11

**Authors:** Samuel Briggs‐Price, Jitendra Mangwani, Alexander Kilcran, Anchal Prasad, Reihaneh Salimian, Seth O'Neill

**Affiliations:** ^1^ University of Leicester School of Healthcare Leicester UK; ^2^ Orthopaedics University Hospitals of Leicester Leicester UK

**Keywords:** Achilles, long term, outcomes, rupture, tendon

## Abstract

**Background:**

Achilles tendon rupture (ATR) is the most common tendon rupture affecting the lower limb. This study investigates Achilles tendon structure, strength and function 1 year or more after ATR.

**Methods:**

This cross‐sectional study recruited individuals ≥ 12 months post ATR. Structure was assessed using ultrasound tissue characterisation (UTC) including cross‐sectional area (CSA), aligned fibrillar structure (AFS), disorganised fibrillar structure (DFS) and echo type percentage. Strength was measured using maximal voluntary isometric contraction (MVIC) plantarflexor testing and the calf raise test. Patient‐reported outcomes included the Achilles tendon rupture score (ATRS), EQ‐5D‐5L and general practice physical activity questionnaire (GPPAQ). Achilles tendon resting angle (ATRA) was used as an indirect measure of tendon elongation. The relationship between outcomes and time since ATR was analysed using linear regression adjusting for age, sex, ethnicity and body mass index (BMI). Between limb comparisons were made using paired t‐tests.

**Findings:**

Sixty participants (mean age 55.2 years and 78.5% male) were assessed at a mean of 6.8 years post ATR. The affected tendon showed a 62% larger cross‐sectional area, with 28.7 mm^2^ (16%) DFS compared to 7.3 mm^2^ (7%) on the nonaffected side (*p* < 0.001). Linear regression showed decreasing AFS with time postinjury (*p* = 0.04); no significant associations were found for CSA or DFS. Significant deficits were observed in plantarflexor strength and function, with MVIC and calf raise work 18% and 40% lower in the affected limb (*p* < 0.001). ATRA indicated tendon elongation in the affected limb of 6.7° (*p* < 0.001). Median ATRS was 83, EQ‐5D index was 0.95 and VAS was 85; 93% were physically active based on the GPPAQ.

**Conclusion:**

Significant structural and functional deficits persist years after ATR, including increased tendon size, fibre disorganisation, reduced strength and tendon elongation. ATRS scores were consistent with previous nonsurgical immobilisation protocol outcomes in the United Kingdom. Longitudinal studies are needed to understand the trajectory of recovery following ATR.

## 1 Introduction

Achilles tendon ruptures (ATRs) are among the most common tendon injuries in physically active individuals, particularly middle‐aged recreational athletes [1]. Recent trends indicate a rising incidence of ATR alongside an increased preference for nonsurgical management strategies [2, 3]. In the United Kingdom (UK), nonsurgical approaches are now the predominant treatment pathway for ATR [4], although there are limited studies assessing individuals following the initial 12 months post ATR.

Understanding tendon healing and structural adaptations following ATR is critical for optimising rehabilitation outcomes. Ultrasound imaging plays a key role in monitoring tendon morphology, with tendon cross‐sectional area (CSA) being a commonly assessed parameter linked to tendon function [5]. Ultrasound tissue characterisation (UTC) offers detailed and quantifiable insights into tendon fibre alignment and quality, exceeding the capabilities of conventional B‐mode ultrasound [6]. Although UTC has been used to characterise tendon structure after ATR [7, 8], there is limited evidence quantifying both tendon CSA and the absolute distribution of organised and disorganised fibres 1 year after injury.

In addition to structural changes, persistent deficits in plantarflexor strength and function are long‐term consequences of ATR [9]. Earlier studies assessing strength have overlooked modern functional bracing and early mobilisation protocols now commonly used in the UK [10]. More recent investigations have begun to incorporate dynamised orthoses [11, 12] but lack plantarflexor strength assessments. Isometric strength testing can provide a safe assessment early in rehabilitation and at long‐term follow‐up, offering consistent insights into strength recovery. Current measures of plantarflexor function have largely been limited to number of calf raise repetitions, an insufficient metric due to variance in height between reps. Recent evidence has called for more quantifiable valid metrics including repetitions and total height raised which allows the calculation of total work performed [13, 14]. This highlights the need for robust and objective strength testing to better understand functional recovery post ATR.

Achilles tendon elongation is another factor to consider when evaluating recovery after ATR as it is associated with end range plantarflexor strength and altered ankle biomechanics [15, 16, 17]. The Achilles tendon resting angle (ATRA) is a valid, reliable and simple indirect measure of tendon length [5, 18], although it has not yet been evaluated after the Leicester Achilles Management Protocol (LAMP). The LAMP represents a modern nonsurgical approach incorporating functional bracing and early weight bearing, measuring the ATRA can offer valuable insights into tendon elongation and help inform future rehabilitation strategies to minimise elongation [19]. Previous work has identified tendon elongation continues to change up to 12 months postinjury [18], highlighting the need to assess elongation at longer‐term follow‐up.

This study aimed to investigate the structural adaptations of the Achilles tendon, plantarflexor strength and function, tendon elongation and patient‐reported outcomes after 1 year or more following nonsurgical management of ATR in the UK.

## 2 Methods

### 2.1 Trial Design

This observational study has a cross‐sectional design. Data were collected from individuals diagnosed with ATR at least 12 months prior to study assessments. Recruitment was completed between December 2024 and April 2025. The study protocol was approved by Yorkshire and The Humber—Leeds West Research Ethics Committee (24/YH/0151). All participants provided written informed consent prior to participation. We adhered to the STROBE guideline for reporting of cross‐sectional studies [20].

### 2.2 Participants

All individuals received a clinical diagnosis of ATR [21] confirmed in a specialist ATR clinic. All participants who had attended the specialist ATR clinic between 2015 and 2023 and had available contact details were sent an invitation to participate (*n* = 361). Participants were routinely managed using the LAMP or Covid‐modified LAMP (CM‐LAMP) protocol consisting of 8 weeks of functional bracing using a Vacoped boot (Table 1) [19]. The CM‐LAMP allowed the patients to alter the boot dynamisation (ankle position and movement range) at home.

**TABLE 1:** LAMP and CM‐LAMP immobilisation protocols.

Time (weeks)	LAMP	Boot^a^
0–4	Locked in 30° plantarflexion	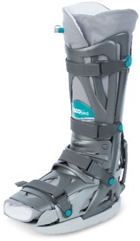
4–6	Dynamised 15°–30° plantarflexion	
6–8	Dynamised 0°–30° plantarflexion	
8	Boot removed	
**CM‐LAMP**		
0–2	Locked in 30° plantarflexion	
2–8	Dynamised range increase 5° per week (e.g., week 2–3 allows 5° from 25° to 30° plantarflexion)	
8	Boot removed	

^a^ VACOped Functional Brace.

### 2.3 Study Procedure

Participants completed assessments once at a single research visit. Participants were not provided their individual findings until they had completed all study outcomes. Demographic data, comorbidities and mechanism of injury were self‐reported by participants.

### 2.4 Study Outcomes

#### 2.4.1 Ultrasound Tissue Characterisation

The UTC SMART system (UTCImaging, Stein, The Netherlands) was used and consisted of conventional ultrasound equipment (multifrequency 5–16 MHzlinear‐array transducer). UTC scans of the Achilles tendon were completed using a standardised protocol with participants prone in a maximal dorsiflexion position [6]. UTC scans were completed for the affected and nonaffected limbs.

Four different echo types are identified using a computer algorithm and represent tendon integrity and fibrillar disorganisation: (I) highly stable, (II) medium stable, (III) highly variable and (IV) constantly low intensity and variable distribution [22]. The intraclass correlation coefficient for intraobserver (0.97–0.99) and interobserver reliability (0.89–0.99) was excellent in previous studies quantifying pathological Achilles tendon structure [23, 24].

**FIGURE 1:**
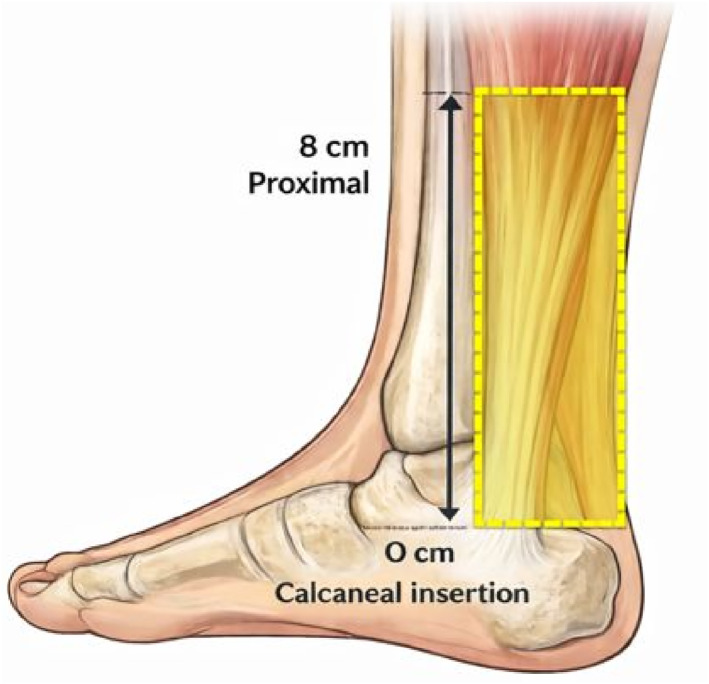
Ultrasound tissue characterisation region of tendon interest.

Assessment and analysis of UTC images adhered to recommended criteria [6]. Any artefacts that could influence analysis were reviewed by a second researcher (SO) to assess whether the image would be excluded. The region of tendon interest was defined as 8 cm from the calcaneus and analysed with a contour interval of 4 mm (20 slices) using a window size of 17 (Figure 1). The interpolation function was used to merge the contour intervals. Tendon CSA was calculated using pixel size and echo type percentage reported as aligned fibrillar structure (AFS) (echo types I and II) and disorganised fibrillar structure (DFS) (echo types III and IV).

#### 2.4.2 Strength Assessments

Maximal voluntary isometric contraction (MVIC) of the plantarflexors was measured using the Fysiometer C‐station (Fysiometer ApS, Denmark) for the affected and nonaffected limbs. Consistent with previous studies, MVIC testing was completed in plantargrade [25]. The testing procedure followed a previously validated method for assessing plantarflexor strength, with the peak from three maximal contractions used as the main outcome [25]. Additional to maximal force output in kilogrammes, MVIC was reported as a strength to bodyweight ratio (MVIC/bodyweight). Limb symmetry index (LSI) was calculated as a percentage for plantarflexor MVIC ((affected limb/nonaffected limb) × 100).

#### 2.4.3 Calf Raise Test

The calf raise test was performed for both limbs using the ‘calf raise app’ following a standardised protocol [26]. The calf raise test for endurance is performed on one leg with the participant standing on a box with an incline of 10°. An audio player with a metronome every 2 s is used to maintain the frequency of 30 heel‐rises per minute. The test is terminated when the patient stopped, could not maintain the frequency or did not perform a sufficient heel‐rise height. The number of calf raise repetitions as well as the peak height of each heel‐rise and the total work were calculated. Limb symmetry index (LSI) was calculated as a percentage for calf raise total work ((affected limb/nonaffected limb) × 100).

#### 2.4.4 Achilles Tendon Resting Angle

Achilles tendon resting angle test is a validated indirect measure of Achilles tendon elongation [18]. The participant is positioned prone on a plinth with knees flexed to 90°. The angle is measured between the long axis of the fibula and the line from the tip of the fibula to the head of the fifth metatarsal.

#### 2.4.5 Patient Reported Outcomes

In addition to the structural integrity and physical function tests, participants also completed patient reported outcome measures.

- Achilles tendon rupture score (ATRS) [27]
- EuroQol‐5 Dimension‐5 Level (EQ‐5D‐5L) [28]
- Global physical activity questionnaire (GPPAQ) [29]

Consistent with previous studies, participants were also asked the questions;

- ‘Do you ever refrain from any activity due to the fear of reinjuring your Achilles tendon?’ [30, 31]
- Are you satisfied with the outcome following your Achilles tendon rupture? [32, 33]
- What was your compliance to the immobilisation boot on a scale of 0–5 (0 = noncompliance and 5 = maximal compliance)?
- What was your compliance to rehabilitation following the boot on a scale of 0–5 (0 = noncompliance and 5 = maximal compliance)?
- If sporting mechanism of injury, how long were you playing sport prior to your injury and have you returned to your previous level of sport? [32]

### 2.5 Data Analysis

Data were analysed using SPSS (V28.0, IBM, New York, USA). Data distribution was assessed for normality using the Shapiro–Wilk test and reported as means or medians with standard deviations (SDs) or interquartile ranges (IQRs). Differences for between limb measures were compared using a paired *t*‐test or Wilcoxon signed‐rank test. If participants reported a contralateral ATR, this was included in the ruptured limb group as an additional ATR. Participants with a contralateral ATR completed the ATRS and additional relevant questions for each affected limb. Fear of reinjury was asked relative to their current behaviour and therefore represented both limbs for individuals with contralateral ATRs.

Linear regression was used to examine the relationship between time post ATR and UTC AFS, DFS and overall CSA. Linear regression was also completed to examine the relationship between time post ATR and ATRS, MVIC and calf raise test total work. We adjusted the analyses for the predefined variables age (continuous), sex (male/female), ethnicity (White British/other ethnicity) and body mass index (BMI) (continuous). Alpha was set at 0.05.

## 3 Results

A total of 60 participants responded to the study invitation and were recruited. Demographic variables, BMI, waist circumference, number of comorbidities, mechanism of injury (sporting/nonsporting) and ethnicities are reported for all participants in Table 2. The median (IQR) time between injury and immobilisation was 0 [2] days. This indicates that participants were immobilised on the day of injury. Time from ATR to entering the bracing protocol was under 2 weeks for all participants.

**TABLE 2:** Participant demographics, BMI, waist circumference, mechanism of injury, time since rupture, ethnicity and number of comorbidities.

		Mean (SD)
Age, years		55.19 (13.76)
Male (%)		51 (78.5%)
Waist circumference, cm		100.21 (12.85)
BMI, kg/m^2^		28.13 (4.24)
No comorbidities, median (IQR)		1 (2)
MOI sporting (%) (*n* = 67)		41 (61.2%)
Time since rupture, years (*n* = 67)		6.8 (2.5)
Ethnicity (%)	White British	37 (61.7%)
	Asian/British Asian	13 (21.7%)
	Black/Black British	6 (10%)
	Other	4 (6.7%)

*Note: N* = 60. Values are given as mean and standard deviation unless specified.

Abbreviation: MOI = mechanism of Injury.

Seven participants reported a previous contralateral ATR, providing 67 ATRs in total in the affected group. Two ATRs were managed surgically (*n* = 1 moved to ATR clinic following ATR surgery out of area, *n* = 1 patient preference for surgical management) and 3 ATRs were immobilised in a cast (*n* = 3 experienced contralateral ruptures with initial ATR prior to LAMP development). All other ATR's (93%, *n* = 62) were managed using the LAMP or CM‐LAMP protocols (LAMP *n* = 51 and CM‐LAMP *n* = 11). One participant reported a previous ATR on the same side.

For participants with a sporting mechanism of injury, 39% reported that this was their first time participating in the sport or they were returning to the sport after a period of at least once month not participating. Following review from a second researcher (SO), four UTC images were excluded from analysis due to heteroscopic ossification preventing adequate capture of the tendon. Another UTC image was excluded for a participant’s nonaffected limb due to ankle jewellery that the patient was unwilling to remove.

**FIGURE 2:**
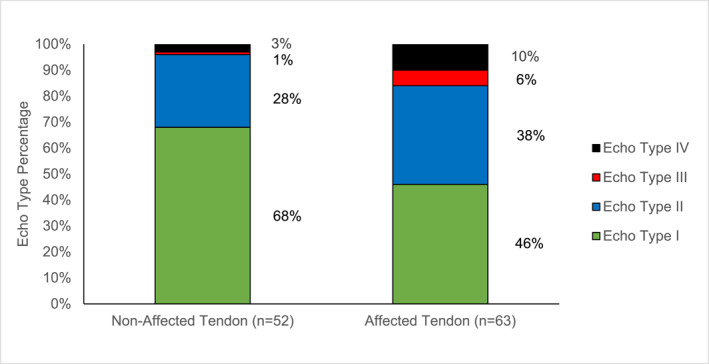
Median ultrasound tissue characterisation echo type percentage between limbs.

Tendon cross‐sectional area, aligned fibrillar structure and disorganised fibrillar structure are provided in Table 3. The affected tendon had a 62% larger tendon cross sectional area than the nonaffected tendon (*p* < 0.001). As a percentage of the cross‐sectional area, the affected tendon had 16% disorganised fibrillar structure and the nonaffected tendon had 4% (*p* < 0.001). UTC echo type percentages for the affected and nonaffected limbs are presented in Figure 2. All UTC echo type percentages were significantly different between limbs (*p* < 0.001).

**TABLE 3:** Ultrasound tissue characterisation, maximal voluntary isometric contraction and calf raise test outcomes for affected and nonaffected limbs.

Variable	Affected limb	Nonaffected limb	Mean difference (95% CI)
Ultrasound tissue characterisation			
Cross‐sectional area, mm^2^	178.31 (49.44)	110.50 (31.77)	67.81*** (54.81–80.81)
Aligned fibrillar structure, mm^2^	149.62 (27.47)	103.20 (38.9)	46.42*** (35.20–57.64)
Disorganised fibrillar structure, mm^2^	28.69 (22.30)	7.29 (7.0)	21.40*** (15.56–27.24)
Maximal voluntary isometric contraction			
MVIC absolute force, kg	83.63 (26.51)	102.00 (28.43)	18.38*** (23.36–13.39)
MVIC normalised to BW, kg/BW	1.01 (0.36)	1.25 (0.46)	0.24*** (0.30−−0.30)
Calf raise test			
Calf raise test repetitions	12.77 (2.11)	19.00 (8.42)	6.23*** (8.31–4.15)
Calf raise test peak height, cm	4.80 (2.11)	6.03 (2.18)	1.23*** (1.71–0.76)
Calf raise test total work, joules	504.80 (382.68)	845.46 (462.43)	315.06*** (219.30–410.83)

*Note:* Ultrasound tissue characterisation, maximal voluntary isometric contraction and calf raise test are presented as means (standard deviations).

Abbreviations: 95%CI = 95% confidence interval, BW = bodyweight, j = joules and MVIC = maximal voluntary isometric contraction.

^***^ *p*<.001.

**FIGURE 3:**
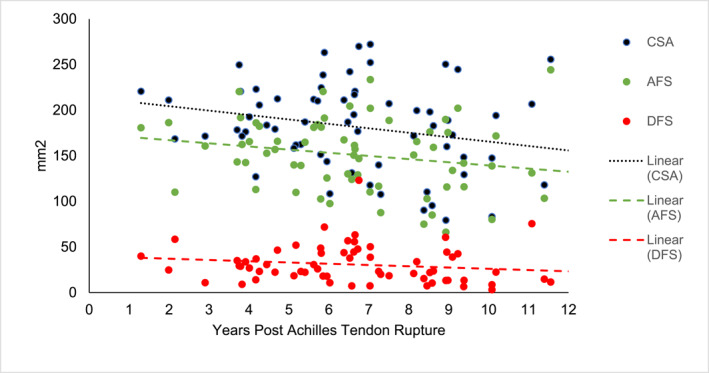
Relationship between cross‐sectional area, aligned fibrillar structure, disorganised fibrillar structure and years since ATR.

The association between days postrupture and total CSA indicated a trend of reducing CSA of 4.75 mm^2^ per year, although this association did not reach statistical significance (*p* = 0.07). There was no significant association between number of days post ATR and DFS. There was a significant association between increasing days post ATR and decreasing AFS (*P* = 0.04). For each year post ATR, a reduction of 4.38 mm^2^ was estimated. Figure 3 illustrates the relationship between CSA, AFS, DFS and years since ATR.

MVIC scores, calf raise test repetitions, peak height and total work scores for the affected and nonaffected limbs are presented in Table 3. MVIC and calf raise test total work LSI demonstrated an 18% and 40% strength deficit in the affected limb compared to the nonaffected limb. ATRA for the affected limb were 54.66 (6.90) and 47.96 (6.12) for the nonaffected limb (Mean difference: 6.70) (95% CI: 4.89–8.51) (*p* < 0.001). Linear regression found no significant association between the number of days post ATR and ATRS, MVIC or calf raise test total work. Additional participant questions are presented in Table 4. ATRS, EQ‐5D‐5L and GPPAQ scores are presented in Table 5. For completeness the mean ATRS was 75, median (IQR) is reported in Table 5 to appropriately represent data distribution. There was no significant difference in ATRS, ATRA or MVIC between the LAMP and CM‐LAMP protocols.

**TABLE 4:** Fear of reinjury, satisfaction with outcome, return to sporting level and compliance to immobilisation protocol and rehabilitation.

Participant question	Yes	No
Pain prior to injury (%)	8 (11.94%)	59 (88.06%)
Fear of reinjury limiting activity (%) (*n* = 60)	30 (50.00%)	30 (50.00%)
Satisfied with outcome (%)	53 (79.1%)	14 (20.9%)
Returned to previous level of sport (%)	27 (40.3%)	40 (59.7%)
Compliance to immobilisation protocol (%)	0/5	0
	1/5	0
	2/5	1 (1.5%)
	3/5	1 (1.5%)
	4/5	10 (14.9%)
	5/5	55 (82.1%)
Compliance to rehabilitation (%)	0/5	0
	1/5	1 (1.5%)
	2/5	2 (3%)
	3/5	9 (13.4%)
	4/5	13 (19.4%)
	5/5	42 (62.7%)

*Note: n* = 67. Fear of reinjury was asked relative to their current behaviour and therefore represented both limbs for individuals with contralateral ATR's (*n* = 60).

Abbreviations: 0 = noncompliance and 5 = maximal compliance.

**TABLE 5:** Patient reported outcomes (ATRS, EQ5D and GPPAQ).

		Median (IQR)
ATRS, *n* = 67		83 (30.0)
EQ‐5D‐5L index		0.95 (0.12)
EQ‐5D‐5L VAS		85 (15)
GPPAQ	Inactive	1 (1.6)
	Moderately inactive	0 (0)
	Moderately active	3 (5%)
	Active	56 (93.3%)

*Note: n* = 60. Values are given as mean and standard deviation.

Abbreviations: ATRS = Achilles tendon rupture score, EQ‐5D = EuroQol‐5 Dimension 5‐Level, VAS = visual analogue scale and GPPAQ = General practice physical activity questionnaire.

## 4 Discussion

This study provides novel insights into persistent tendon structural deficits post ATR. After a minimum of 1 year, the affected tendon continues to have a significantly larger CSA and a higher proportion of DFS compared to the nonaffected limb. Despite a significant reduction in AFS, the trend towards CSA and DFS reducing over time did not reach statistical significance. Reasons for this may include; insufficient sample size to detect statistical difference, inability for the tendon to adequately remodel or inadequate functional recovery possibly due to incomplete rehabilitation limiting adaptive stimulus. The tissue disorganisation in the affected limb was also identified by significantly greater percentage of disorganised echo type percentage in the affected tendon. Previous studies combining surgical and nonsurgical management have reported stable UTC echo type percentages over the initial 12 months post ATR [34]. Participants in this study presented with notably less disorganised echo types (types 3 and 4) at 6.8 years than reported by Dams (2021) at 12 months. This may be due to differences in age or ATR management strategies between studies. However, as this study also assessed CSA, DFS and AFS, it was able to identify a relationship between time post ATR and tendon structure. Future longitudinal studies should consider longer follow‐up durations as changes in tendon structure may continue after the initial 12 months post ATR.

Participants exhibited significant deficits in plantarflexor strength and endurance. Consistent with prior studies, MVIC values and calf raise metrics (total work and height) were significantly lower in the affected limb [15, 17, 35, 36]. Based on LSI, the plantarflexor deficits were more severe during repeated isotonic contraction using the calf raise test. This likely reflects the altered neuromuscular properties and tendon elongation that occur post ATR. The ATRA was significantly greater in the affected limb, suggesting tendon elongation at comparable levels to previous surgical protocols [18]. This elongation may primarily drive the ongoing peak height deficit seen during the calf raise test [15]. However, both limbs were below the normative value of 24–25 calf raise repetitions reported in individuals of comparable age, BMI and activity level [13, 14]. Insufficient plantarflexor strength in the nonaffected limb may reduce the buffering effect of the muscle on the Achilles tendon [37]. Therefore, exposing the Achilles tendon to increased stress, potentially providing an insight into the high rates of contralateral rupture seen in this study (12%) and the wider ATR population [38].

The median ATRS score was 83, although the mean ATRS was consistent with previous studies evaluating the LAMP and CM‐LAMP protocols at 12‐ and 23‐month post ATR [19, 39]. There was no association between time post ATR and ATRS; this indicates that higher scores may require an improvement in structure or function as time was insufficient to improve patient reported function. Despite this, 79% of participants expressed satisfaction with their recovery and 93% were classified as physically active. However, 50% of participants reported fear of reinjury limiting activity, and only 40% returned to their previous level of sport. These factors are likely related as fear of reinjury has been reported to influence return to sport decision making following ATR [30, 40]. These findings suggest a disconnection between objective impairments and perception of recovery reported subjectively, possibly due to adaptive strategies or modified expectations of recovery.

### 4.1 Study Limitations

The cross‐sectional design of the study limits the ability to infer causation. Although associations between tendon structure, function and time since rupture were explored, longitudinal data are required to understand the trajectory of these changes. Four UTC scans were excluded from analysis due to heterotopic ossification, which impeded the UTC quantification algorithm. This phenomenon may be more prevalent in individuals assessed beyond 12 months postinjury, potentially reflecting a complication of healing. No power calculation was conducted prior to recruitment, which may affect the findings from statistical testing. Although significant differences were observed across several outcomes, the study may be underpowered to detect associations such as changes seen in tendon CSA and DFS. A further consideration is the inclusion of different management and immobilisation strategies included in this study; this reflects routine clinical management in this NHS service. Future studies should include adequately powered, prospective comparisons between immobilisation protocols.

## 5 Conclusion

After a minimum of 1 year following an ATR, individuals present with persistent and significant structural, strength and functional deficits. These include significantly greater CSA, increased DFS, reduced plantarflexor strength (MVIC and Calf raise test) and tendon elongation. Although most participants are satisfied with the outcome, only 40% returned to their previous level of sport and 50% reported a fear of reinjury limiting activity. These findings highlight the need to explore recovery trajectories and develop strategies to improve long‐term outcomes after an ATR.

## Author Contributions

**Samuel Briggs‐Price:** conceptualisation, methodology, investigation, formal analysis, data curation, writing – original draft, writing – review and editing. **Jitendra Mangwani:** supervision, writing – review and editing. **Alexander Kilcran:** investigation, formal analysis, writing – review and editing. **Anchal Prasad:** investigation, writing – review and editing. **Reihaneh Salimian:** investigation, formal analysis, writing – review and editing. **Seth O'Neill:** supervision, formal analysis, writing – review and editing.

## Funding

This study was completed as part of a Health Sciences PhD at the University of Leicester School of Healthcare funded by the University of Leicester Future 100's scheme.

## Ethics Statement

This study was granted ethical approval by Yorkshire and The Humber—Leeds West Research Ethics Committee (24/YH/0151).

## Conflicts of Interest

The authors declare no conflicts of interest.

## Supporting information


Supporting Information S1

## Data Availability

All relevant data are available at 10.25392/leicester.data.30050305.
